# Occipital Condyle Fracture: A Case Report of a Typically Stable Fracture That Required Surgical Treatment

**DOI:** 10.1155/2018/2809546

**Published:** 2018-11-15

**Authors:** Takeshi Suzuki, Satoshi Maki, Masaaki Aramomi, Tomonori Yamauchi, Manato Horii, Koui Kawamura, Hiroshi Sugiyama, Seiji Ohtori

**Affiliations:** ^1^Department of Orthopaedic Surgery, Asahi General Hospital, i1326 Asahi, Chiba 289-2511, Japan; ^2^Department of Orthopaedic Surgery, Chiba University, Graduate School of Medicine, 1-8-1 Inohana Chuou-ku, Chiba, Chiba 260-8670, Japan

## Abstract

An occipital condyle fracture (OCF) is a relatively rare trauma that is now increasingly diagnosed because of the wide availability of computed tomography. For nondisplaced OCFs, conservative treatment is generally recommended, and there is no previous report of a nondisplaced OCF requiring surgery. We report a patient who had a nondisplaced OCF with craniocervical misalignment (a condyle-C1 interval > 2.0 mm) and C1-C2 translation treated with a halo vest and occipitocervical fusion surgery. An 87-year-old Asian woman fell from a 4-meter height and hit her head. She was transferred to our emergency room. Computed tomography revealed a nondisplaced impaction OCF with a 2.5 mm occipital condyle-C1 interval and a 5 mm C1-C2 translation. The fracture pattern was considered stable. However, since craniocervical misalignment and C1-C2 translation were present, the patient was placed in a halo device, and we reduced the occipitoatlantoaxial joint, adjusting the halo ring position preoperatively. Confirming reduction of the atlantooccipital facet joint and the atlantoaxial joint by computed tomography, we performed an occipitocervical fusion. This is the first report of a nondisplaced OCF with craniocervical misalignment and C1-C2 translation that required surgical treatment. Clinicians should be aware of craniocervical misalignment and atlantoaxial instability even in Tuli type 1 OCFs.

## 1. Introduction

An occipital condyle fracture (OCF) was once a relatively rare traumatic injury, but is now increasingly diagnosed because of the wide availability of computed tomography [1, 2]. Tuli et al. reported a classification for OCF based on the stability of the O-C1-C2 complex [3]. In their classification, conservative treatment is recommended for type 1 fractures, which have a nondisplaced fracture or impaction fracture; however, a halo vest or surgical treatment is often recommended for type 2B fractures, which have a displaced fracture with ligamentous injury. Although there are several case reports on type 1 OCF, there is no report of a case that required surgical treatment. Here, we report the case of a patient who had a type 1 OCF with craniocervical misalignment and C1-C2 translation that was treated with a halo vest followed by occipitocervical fusion surgery. To the best of our knowledge, this is the first report of a type 1 OCF with craniocervical misalignment and C1-C2 translation that necessitated surgery.

## 2. Case Presentation

**Figure 1:**
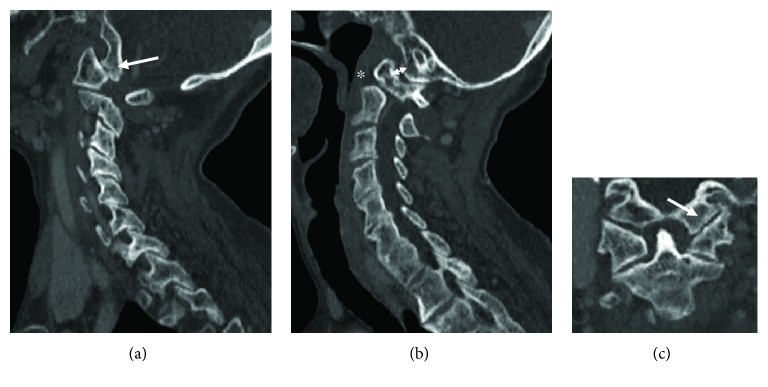
Left parasagittal (a) and coronal (c) CT scan images show a right nondisplaced impaction occipital condyle fracture (arrow), classified as Anderson and Montesano type I and Tuli type 1. Right parasagittal CT image (b) shows an occipital condyle-C1 interval of 2.5 mm (double-headed arrow) and a 5 mm translation of C1-C2 (asterisk).

**Figure 2:**
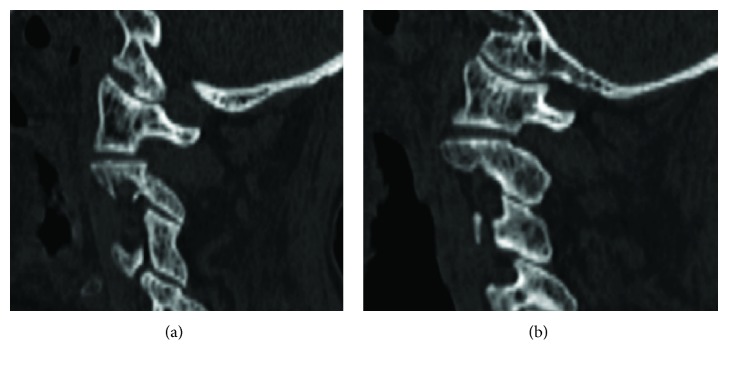
Left (a) and right (b) parasagittal CT scan images demonstrate complete reduction of both the atlantooccipital joint and the atlantoaxial joint.

**Figure 3:**
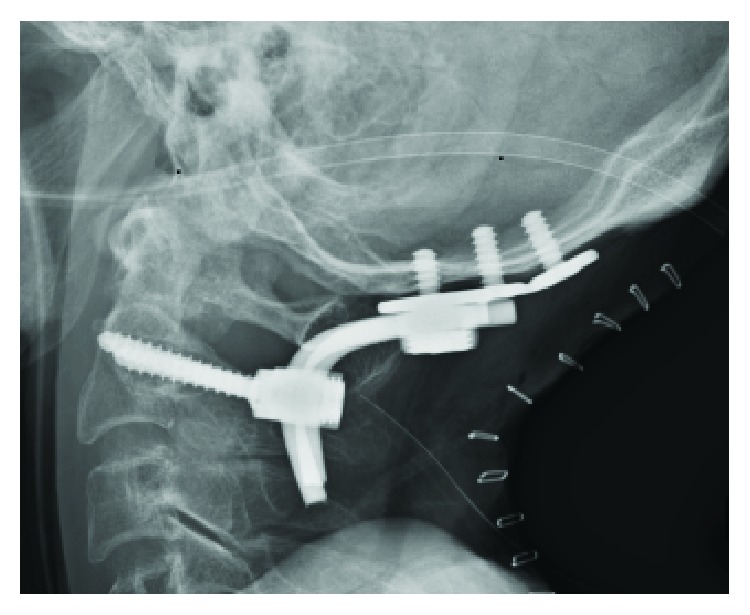
Postoperative radiograph shows occipitocervical fusion achieved by an occipital plate and bilateral C2 pedicle screws.

An 87-year-old Asian woman who fell from a 4-meter height and hit her head was transferred to our emergency room. It was difficult to conduct a detailed neurological examination due to her severe dementia, but she had no obvious neurological symptoms upon arrival at our hospital. Computed tomography of her cervical spine showed a left nondisplaced impaction OCF with an occipital condyle-C1 interval of 2.5 mm and a 5 mm translation of C1-C2 (Figure 1). The fracture pattern itself, classified as Anderson and Montesano type 1 and Tuli type 1, was considered stable. However, as a craniocervical misalignment and C1-C2 translation were present, the patient was placed in a halo device temporarily before surgery. We decided to perform reduction prior to surgery; thus, while we applied rotational traction force, we adjusted the halo ring position. After reduction, computed tomography was performed and we confirmed the complete reduction of both the atlantooccipital joint and the atlantoaxial joint (Figure 2). We proceeded to internal segmental fixation with an occipital bone plate and bilateral pedicle screws for C2 (Figure 3). An autologous iliac crest bone graft was used in an augmented posterior fusion. The halo vest was removed immediately after surgery. The patient's clinical status improved, and she was discharged to a rehabilitation facility.

## 3. Discussion

Nondisplaced or impacted OCF is generally treated conservatively. However, our patient had craniocervical misalignment and a C1-C2 translation that required surgery. Thus, her fracture pattern was exceptional for a Tuli type 1 fracture, for which conservative treatment is recommended. Anderson and Montesano first reported a classification for OCFs in 1988 [4]. Tuli et al. published the second classification for OCFs in 1997, which was aimed at being an efficient treatment guidance; they broadened the definition of instability to include the integrity not only of the atlantooccipital joint but also of the atlantoaxial joint [3]. The following definitions were provided: type 1, nondisplaced OCF; type 2A, displaced OCF with intact ligaments; and type 2B, displaced OCF with radiographic evidence of craniocervical junction instability. Craniocervical junction instability was defined as the presence of 8 markers of instability: (1) >8° of axial rotation of the atlantooccipital joint; (2) >1 mm of atlantooccipital joint translation; (3) >7 mm of overhang of C1 on C2; (4) >45° of axial rotation of C1-C2; (5) >4 mm of C1-C2 translation; (6) <13 mm distance between the posterior body of C2 to the posterior ring of C1; or (7) an avulsed transverse ligament; or alternatively, (8) the finding on MR imaging of evidence of ligamentous disruption. Tuli et al. hypothesized that their classification can guide treatment: type 1 OCF does not require immobilization; type 2A OCF should be treated with a rigid collar; and type 2B OCF requires surgical fixation or a halo vest. Although their classification can be a good reference for the management of OCFs, it is cumbersome, as highlighted by Hanson et al. and Aulino et al. in their retrospective reviews; the definition of fracture displacement is equivocal and the criteria for instability is somewhat arbitrary [5, 6]. For other recommendations for surgical management of OCFs, Hanson et al. suggested that a CT finding of bilateral occipitoatlantoaxial joint complex injury (defined as either bilateral OCF or unilateral OCF with contralateral widening of the atlantooccipital (>2 mm) or atlantoaxial (>3 mm) joint) be used as a marker for instability and surgical indication [5]. Maserati et al. proposed criteria to simplify identification of the Tuli type 2b OCF. They defined craniocervical misalignment as a condyle-C1 interval > 2.0 mm on reconstructed CT images [7]. Surgical stabilization is recommended for patients with an OCF resulting in craniocervical misalignment. Their other proposed indication for surgery is neural element compression by fracture fragments or an associated hematoma. Although there were no patients with Anderson and Montesano type 1 or Tuli type 1 OCF who underwent surgery in any previous case series or case report, our patient met Hanson's and Maserati's criteria for surgery but not Tuli's criteria. Most of the reports on Tuli's type 1 OCF showed an OCF in the medial part, while our patient had an OCF in the lateral part. Although the details are unknown, axial and rotational forces seem to have been applied to the head.

Reducing the atlantooccipital joint and the atlantoaxial joint in a halo device and confirming reduction by CT prior to surgery could obviate the need for intraoperative reduction and enable in situ fusion. We considered that confirming reduction of the atlantooccipital joint by intraoperative fluoroscopy would be extremely difficult since 93.3% of OCFs were overlooked in a plain radiogram [8]. Thus, preoperative reduction of the atlantooccipital joint and preoperative confirmation of the reduction by CT was an efficient way to restore alignment of the occipitoatlantoaxial joint complex.

This is the first report of a Tuli type 1 OCF with craniocervical misalignment and C1-C2 translation that required surgical treatment. Although it is unusual, this case illustrates that an occipitoatlantoaxial joint complex injury can occur in Tuli type 1 OCFs. Clinicians should be aware of craniocervical misalignment and atlantoaxial instability even in a Tuli type 1 OCF.
